# Supplementary material for: Primary care clinical management following self-harm during the first wave of COVID-19 in the UK: population-based cohort study
Source: BMJ Open. 2022 Feb 14;12(2):e052613. doi: 10.1136/bmjopen-2021-052613 (PMC8844953; doi:10.1136/bmjopen-2021-052613)
Supplement: Author's manuscript [file bmjopen-2021-052613.draft_revisions.pdf]

BMJ Open is committed to open peer review. As part of this commitment we make the peer review history of every article we publish publicly available.

When an article is published we post the peer reviewers' comments and the authors' responses online. We also post the versions of the paper that were used during peer review. These are the versions that the peer review comments apply to.

The versions of the paper that follow are the versions that were submitted during the peer review process. They are not the versions of record or the final published versions. They should not be cited or distributed as the published version of this manuscript.

BMJ Open is an open access journal and the full, final, typeset and author-corrected version of record of the manuscript is available on our site with no access controls, subscription charges or pay-per-view fees (http://bmjopen.bmj.com).

If you have any questions on BMJ Open's open peer review process please email info.bmjopen@bmj.com

BMJ Open

Primary care clinical management following self-harm during the first wave of COVID-19 in the UK: population-based cohort study

Journal:	*BMJ Open*
Manuscript ID	bmjopen-2021-052613
Article Type:	Original research
Date Submitted by the Author:	23-Apr-2021
Complete List of Authors:	Steeg, Sarah; The University of Manchester Faculty of Biology Medicine and Health<br>Carr, M<br>Trefan, Laszlo<br>Ashcroft, Darren; University of Manchester<br>Kapur, Navneet; University of Manchester, Centre for suicide prevention<br>Nielsen, Emma; University of Nottingham<br>McMillan, Brian; The University of Manchester, Centre for Primary Care and Health Services Research;<br>Webb, Roger; The University of Manchester, School of Community Based Medicine
Keywords:	PRIMARY CARE, Suicide & self-harm < PSYCHIATRY, PUBLIC HEALTH, COVID-19

SCHOLARONE™
Manuscripts

I, the Submitting Author has the right to grant and does grant on behalf of all authors of the Work (as defined in the below author licence), an exclusive licence and/or a non-exclusive licence for contributions from authors who are: i) UK Crown employees; ii) where BMJ has agreed a CC-BY licence shall apply, and/or iii) in accordance with the terms applicable for US Federal Government officers or employees acting as part of their official duties; on a worldwide, perpetual, irrevocable, royalty-free basis to BMJ Publishing Group Ltd ("BMJ") its licensees and where the relevant Journal is co-owned by BMJ to the co-owners of the Journal, to publish the Work in this journal and any other BMJ products and to exploit all rights, as set out in our licence.

The Submitting Author accepts and understands that any supply made under these terms is made by BMJ to the Submitting Author unless you are acting as an employee on behalf of your employer or a postgraduate student of an affiliated institution which is paying any applicable article publishing charge ("APC") for Open Access articles. Where the Submitting Author wishes to make the Work available on an Open Access basis (and intends to pay the relevant APC), the terms of reuse of such Open Access shall be governed by a Creative Commons licence – details of these licences and which Creative Commons licence will apply to this Work are set out in our licence referred to above.

Other than as permitted in any relevant BMJ Author's Self Archiving Policies, I confirm this Work has not been accepted for publication elsewhere, is not being considered for publication elsewhere and does not duplicate material already published. I confirm all authors consent to publication of this Work and authorise the granting of this licence.

For peer review only

Primary care clinical management following self-harm during the first wave of COVID-19 in the UK: population-based cohort study

Sarah Steeg*[1,2], Matthew J Carr*[1, 2-4], Laszlo Trefan, Darren M Ashcroft [1, 2-4], Nav Kapur [1,2,4,5], Emma Nielsen [6], Brian McMillan[1, 7], Roger T Webb [1,2,4].

*Joint first authors

Sarah Steeg, PhD, Presidential Research Fellow

Matthew J Carr, PhD, Research Fellow

Laszlo Trefan, PhD, Research Fellow

Darren M Ashcroft, PhD FRPharmS, Professor of Pharmacoepidemiology

Nav Kapur, FRCPsych, MD, Professor of Psychiatry and Population Health

Emma Nielsen, BSc, Research Fellow

Brian McMillan, PhD, Clinical Lecturer

Roger T Webb, PhD, Professor of Mental Health Epidemiology

[1] Centre for Mental Health and Safety, Manchester Academic Health Science Centre, University of Manchester, Manchester, UK.

[2] Manchester Academic Health Science Centre, University of Manchester, Manchester, UK.

[3] Centre for Pharmacoepidemiology and Drug Safety, National Institute for Health Research Greater Manchester Patient Safety Translational Research Centre, University of Manchester, Manchester, UK.

[4] National Institute for Health Research Greater Manchester Patient Safety Translational Research Centre, University of Manchester, Manchester, UK.

[5] Greater Manchester Mental Health NHS Foundation Trust, Manchester, UK.

[6] Self-harm Research Group, School of Psychology, University of Nottingham, UK.

[7] Centre for Primary Care and Health Services Research, University of Manchester, Manchester, UK.

Corresponding author: Sarah Steeg, sarah.steeg@manchester.ac.uk; 2.308 Jean McFarlane Building, University of Manchester, Oxford Road, Manchester, M13 9PL, UK; Tel +44(0)161 275 0718, Fax: +44 (0)161 275 0716

For peer review only - http://bmjopen.bmj.com/site/about/guidelines.xhtml

Abstract

Objectives

A substantial reduction self-harm recorded in primary care occurred during the first wave of COVID-19 but effects on primary care management of self-harm are unknown. Our objectives were to examine the impact of COVID-19 on clinical management within three months of an episode of self-harm.

Design

Retrospective cohort study.

Setting

UK primary care.

Participants

4,238 patients with an index episode of self-harm recorded in UK primary care during the COVID-19 first-wave period (10th March-10th June 2020) compared to 48,739 patients in a pre-pandemic comparison period (10th March-10th June, 2010-2019).

Outcome measures

Using data from the UK Clinical Practice Research Datalink we compared cohorts of patients with an index self-harm episode recorded during the pre-pandemic period versus the COVID-19 first-wave period. Patients were followed up for three months to capture subsequent psychotropic medication prescribing, GP/practice nurse consultation and referral to mental health services. We examined differences by gender, age group and Index of Multiple Deprivation (IMD) quintile.

Results

Similar proportions were prescribed psychotropic medication within 3 months in the pre-pandemic (54.0%) and COVID-19 first-wave (54.9%) cohorts. Likelihood of having at least one GP/practice nurse consultation was broadly similar (83.2% vs. 80.3% in the COVID-19 cohort). The proportion of patients referred to mental health services in the COVID-19 cohort (3.4%) was around half of that in the pre-pandemic cohort (6.5%).

Conclusions

For peer review only

Despite the challenges experienced by primary healthcare teams during the initial COVID-19 wave, prescribing and consultation patterns following self-harm were broadly similar to pre-pandemic levels. However, the reduced likelihood of referral to mental health services warrants attention. Accessible outpatient and community services for people who have self-harmed are required as the COVID-19 crisis recedes and the population faces new challenges to mental health.

Keywords: general practice; self-harm; mental health; COVID-19; psychotropic drugs; epidemiology.

Strengths and limitations of this study

- First study examining effects of Covid-19 on primary care management following self-harm in the UK
- Findings are based on a large number of general practices across the UK, using data from the Clinical Practice Research Datalink
- Data broadly representative of geographical coverage, area-level deprivation, age, and sex in England
- Data accuracy is determined by the quality of the information inputted by contributing practices.
- We were unable to examine referrals to mental health services in England.

For peer review only - http://bmjopen.bmj.com/site/about/guidelines.xhtml

Introduction

Prompt clinical intervention and follow-up is recommended for people who have recently self-harmed, in part due to their increased risks of suicide. [1] Self-harm includes intentional self-poisoning and self-injury and can involve varying degrees of suicidal intent. [2] All episodes of self-harm should be followed by comprehensive mental health assessment to identify psychosocial needs and address risks of further self-harm and suicide. [1] This has become more challenging due to the disruption to UK health services caused by the COVID-19 pandemic. Fluctuations in infection rates, lockdown restrictions and public health messaging has had a significant impact on the numbers of people accessing primary care services, with considerable reductions in presentation rates found for a number of physical and mental health conditions, [3] including self-harm. [4] The pandemic and its pervasive impacts on everyday life has also had a detrimental impact on the mental health of the population [5] and the first wave of COVID-19 and lockdown may have led to an increase in the prevalence of suicidal ideation. [6] This unique combination of factors has led to a shortfall in the numbers of people receiving support from healthcare services after harming themselves.

In April 2020, during the first UK COVID-19 lockdown, rates of primary care-recorded incident self-harm in the UK were 38% lower than expected based on trends that occurred during the previous ten years. [4] While rates of help seeking gradually returned towards expected levels through the subsequent months up to September 2020, further regional COVID-19 containment restrictions and national lockdowns from autumn 2020 into winter 2021 are likely to have additionally affected rates of help seeking. Some people sought help from alternative sources whilst the UK was in its first lockdown; for example, some mental health charities reported increases in demand for services such as helplines. [7] One study in the US reported emergency hospital presentations following suicide attempts had increased to higher than pre-pandemic levels following an initial reduction, [8] suggesting that observed reductions in help seeking in primary care did not necessarily reflect population need.

Primary care settings provide vital support for people who have self-harmed. A recent study found that 26% of people sought help from their general practitioner (GP) in the week prior to suicide, with self-harm a common reason for contact.[9] Previous research found that 15% of patients with an episode of self-harm recorded in primary care were referred to mental health services from their GP within a year. [10] A recent report found that, among people referred to mental health services, their GP was the most common referral route. [11] However, this report highlighted many barriers that exist for people accessing support following self-harm. [11] GPs, as well as patients, have reported

struggling to find appropriate self-harm services, with limited referral options and shortages in community services identified. [12]

It is unknown how clinical management following self-harm has been affected by the COVID-19 pandemic. Although GPs continued to offer consultations to patients throughout the first wave in the spring of 2020, with most consultations taking place remotely, [13] recently conducted research found lower rates of GP referral to mental health services following primary care-recorded common mental illnesses and self-harm episodes. [4] The degree to which primary care management of people who had self-harmed was impacted during the first COVID-19 wave is unknown. This is an important research question, because self-harm is a key risk factor for suicide and requires specific timely intervention. [14]

In the UK, a nationwide lockdown was imposed on 23rd March 2020, with public health messaging to encourage people to avoid contact with others announced the week before this. [15] We aimed to examine clinical management of self-harm during the three months following the beginning of the UK COVID-19 containment measures and national lockdown, using data from a pre-pandemic comparison period to examine effects of COVID-19. Our specific objectives were to:

review only

For peer review only - http://bmjopen.bmj.com/site/about/guidelines.xhtml

(i) Identify two cohorts of patients presenting with an index episode of self-harm, comprising pre-pandemic versus COVID-19 first-wave time periods.

(ii) Estimate the likelihood of receiving a new psychotropic medication prescription (by drug type), frequency of subsequent GP or practice nurse consultations, and referral to mental health services within 3 months of the index self-harm episode.

(iii) Estimate rate ratios for frequency of psychotropic medication prescribing, referral to mental health services and GP or practice nurse consultation (by face-to-face and telephone) within three months of the index episode, between the COVID-19 and pre-pandemic comparison cohorts.

Methods

Study design, data sources, and participants

We conducted a prospective cohort study using anonymised primary care data from the Clinical Practice Research Datalink (CPRD) Aurum and GOLD databases. [16 17] The Aurum database includes general practices based in England that contribute data using the EMIS® clinical system. The GOLD database is extracted from the Vision® system, with most of its contributing general practices based in Northern Ireland, Scotland and Wales. The Aurum database is broadly representative of geographical coverage, area-level deprivation, age, and sex distributions of the population of England, [16] whist GOLD is broadly representative of the sociodemographic profile of the whole UK population. [17] To avoid including duplicate general practices in the Aurum and GOLD databases, we excluded English practices in our analyses of GOLD data. The CPRD includes information on patient demographics, consultations, symptoms, diagnoses, medication prescriptions and referrals to secondary care. We also obtained information on IMD linked at general practice level. [18] The IMD is a composite measure of the relative deprivation of an area derived from several domains including income, employment and health. [19]

Analysis was conducted on pooled Aurum and GOLD data. We compared two cohorts of patients: (i) those with an index self-harm episode recorded in a pre-pandemic comparison period (between 10th March to 10th June, 2010-2019) and those with an index self-harm episode recorded in the COVID-19 first-wave period (10th March to 10th June 2020). Patients in each cohort were followed up for three months to capture psychotropic medication prescribing, referral to mental health services (available in GOLD only) and GP or practice nurse consultations. To be included in either of the two study cohorts, patients must have been aged 10 years or older and registered with a contributing practice, deemed by the CPRD as providing up-to-standard data, for at least one year prior to the date of the index self-harm episode. Patients with less than 3 months of follow-up time in the CPRD were excluded from our analyses. The cohorts were restricted to patients with records that were deemed acceptable by the CPRD for research purposes, which excluded

For peer review only

patients with missing data on sex or age. IMD data were missing for 9.2% of the pre-pandemic comparison cohort and 10.3% of the COVID-19 first-wave cohort (Table 1).

The study was approved by the Independent Scientific Advisory Committee (protocol number 20_001RA2).

Exposures, outcomes and covariates

Index episodes of self-harm were identified from Read, SNOMED and EMIS codes. To identify individuals' first record of self-harm, we applied a retrospective analysis period during which a patient was required to have been registered at the practice for at least 1 year before an incident episode. We examined any psychotropic medication and specific psychotropic medication types (https://clinicalcodes.rss.mhs.man.ac.uk/medcodes/article/173/). Mode of consultation was grouped into face-to-face and video/telephone consultations, with a GP or practice nurse. Information on referral to mental health services was only available in the GOLD data. Two fields were used to identify referrals: a 'psychiatry' code in the Family Health Services Authority variable and codes of 'mental illness', 'child and adolescent psychiatry', 'forensic psychiatry', 'psychotherapy', 'old age psychiatry', 'clinical psychology', 'adult psychiatry' and 'community psychiatric nurse' in the National Health Service specialty field. [10] We combined information from both the FHSA and NHS fields to construct a binary specialist mental health services referral indicator. All code lists were verified by senior clinical academics as part of a previous study [4] and are available online (https://clinicalcodes.rss.mhs.man.ac.uk/medcodes/article/173/). We examined frequencies and rate ratios between the pre-pandemic and COVID-19 first-wave cohorts by gender, age group (10-24, 25-64 and 65 and older) and practice-level IMD quintile.

Analysis

Frequencies of psychotropic medication prescribing, GP/nurse consultation and referral to mental health services during the antecedent period were estimated and compared to observed values during the COVID-19 first-wave period. The modelling was conducted using modified Poisson regression in a generalised linear modelling framework with a log-link function and a robust variance estimator with analyses stratified by gender, age group and practice-level IMD quintile. This study was conducted in accordance with REporting of studies Conducted using Observational Routinely-collected health Data guidance (RECORD; Table S1). [20]

Patient and public involvement

A panel of four service users and carers with lived experience of health services following self-harm collaborated with the research team to plan the study and interpret results.

For peer review only - http://bmjopen.bmj.com/site/about/guidelines.xhtml

Results

A total of 48,739 patients had an index episode of self-harm recorded in the UK during the pre-pandemic comparison period and 4,238 were recorded in the COVID-19 first-wave cohort (Table 1). The gender, age and deprivation profiles of the two cohorts were broadly similar, with the majority of recorded self-harm episodes by women and more self-harm episodes recorded in practices in areas of higher deprivation.

Just over half of patients in the pre-pandemic (54.0%) and COVID-19 first-wave (54.9%) cohorts were prescribed psychotropic medication within three months of the index self-harm episode (Table 2). The likelihoods of receiving such treatment were broadly similar across gender and deprivation quintiles. Among patients aged 10-24 years, those in the COVID-19 first-wave cohort were more likely to be prescribed psychotropic medication (ratio 1.14, 95% CI 1.07 to 1.22). Considering prescriptions for antidepressant medication specifically, probabilities were broadly similar in the pre-pandemic and COVID-19 first-wave cohorts, though they were higher among young people aged 10-24 years in the COVID-19 first-wave cohort (ratio 1.18, CI 1.10 to 1.26). Details on prescribing of antipsychotic, anxiolytic/hypnotic, mood stabilisers and stimulants are in supplementary Table S2.

Unsurprisingly, the likelihood of patients receiving a remote GP/practice nurse consultation within 3 months of a self-harm episode was higher in the COVID-19 ‘first-wave’ cohort (67.7%) than in the pre-pandemic comparison cohort (32.3%, ratio 2.10, CI 2.05 to 2.15) (Table 3). Although the overall likelihood of having a GP/practice nurse consultation was slightly lower in the COVID-19 cohort (80.3%) than in the pre-pandemic comparison cohort (83.2%), ratio 0.97, CI 0.96 to 0.98, this pattern did not apply to all demographic groups. Men, patients aged 65 years and over and those registered with practices in the two most deprived quintiles were equally likely to have had a GP/practice nurse consultation in the pre-pandemic and COVID-19 first-wave cohorts.

Information on primary care referrals to mental health services was only available in the GOLD dataset (representing practices in Northern Ireland, Scotland and Wales). Overall, 3.4% of patients (31/910) were referred to mental health services in the COVID-19 first-wave cohort, around half of that in the pre-pandemic cohort (6.5%, ratio 0.53, CI 0.37 to 0.75) (Table 4). The reduction in likelihood of being referred to mental health servicers was greater among women (ratio 0.34, CI 0.19 to 0.60) than for men (0.83, CI 0.53 to 1.30), p value for effect modification by gender = 0.016). The fall in probability of being referred also appeared to be greater

for patients aged 10-24 years (ratio 0.33, CI 0.17 to 0.66). However, the numbers of patients following stratification by referral status and age group was not sufficiently powered to conduct formal tests for interaction effects in these groups.

For peer review only

Discussion

Summary

Pre-pandemic and COVID-19 first-wave cohorts of patients with an index episode of self-harm recorded in primary care had similar gender, age and deprivation profiles. Similar proportions were prescribed psychotropic medication within three months of their index self-harm episode - just over half in both the pre-pandemic and COVID-19 first-wave cohorts. However, patients aged 10-24 years in the COVID-19 first-wave cohort were more likely to be prescribed psychotropic medication than in the preceding years. Overall, the likelihood of having at least one GP/practice nurse consultation was slightly lower in the COVID-19 first-wave cohort, although there was no such difference observed among men, patients aged 65 years and over, and those registered with practices located in more deprived areas. The proportion of patients referred to mental health services (in Northern Ireland, Scotland and Wales) in the COVID-19 first-wave cohort was around half of that in the pre-pandemic comparison cohort with larger deficits in referral likelihood observed among women.

Strengths and limitations

The main strength of our study is the broadly representative data source that included a large number of general practices across the UK. CPRD Aurum is broadly representative of geographical coverage, area-level deprivation, age, and sex in England [16] while CPRD GOLD dataset is broadly representative of the UK age and sex profile.[17] This enables us to make inferences at national level about how the pandemic affected primary care clinical management of patients who have self-harmed. However, there are some limitations in utilising anonymised primary care records. The data are extracted from GP information systems and their accuracy is determined by the quality of the information inputted by contributing practices. Some of the self-harm episodes recorded in primary care would have been emergency department presentations that were subsequently added to the patient's primary care record. Future research using linked Hospital Episode Statistics will enable separate examination of emergency department self-harm presentations. Finally, we were unable to examine referrals to mental health services in England due to limitations to the CPRD Aurum dataset.

Comparison with existing literature

Previous research found that reductions in help-seeking were greatest among patients registered at practices located in more deprived areas.[4] Our study found that patients at practices in the two most deprived quintiles who did seek help had similar rates of psychotropic medication prescribing as those at practices in less deprived areas, in both the pre-pandemic comparison and COVID-19 first-wave cohorts. Similarly, the abrupt switch to remote consultations once the COVID-19 crisis had commenced occurred to a similar degree at practices across the practice population deprivation

For peer review only - http://bmjopen.bmj.com/site/about/guidelines.xhtml

spectrum. This suggests that for people who did seek help, there was no exacerbation of existing inequalities due to COVD-19 in these particular aspects of primary care clinical management.

However, likelihood of referral to mental health services was lower during the first wave of COVID-19 than during the pre-pandemic comparison period, particularly for women. Younger people may also have been less likely to receive a referral for mental health services (though our findings lacked statistical power to infer this with certainty). Both of these groups have been found to have been particularly negatively affected by the pandemic. Individuals aged 18-29 years reported greater increases in suicidal ideation over the first six weeks of the UK's lockdown than other groups, [6] while women and younger people were found to have the greatest deteriorations in mental health. [5] Furthermore, these groups were previously identified as having the greatest reductions in help seeking for mental illness and self-harm during April 2020.[4]

Implications for research and practice

Evidence shows the number of people seeking mental health help, including for self-harm, from non-NHS services such as via digital platforms and helplines increased during the second quarter of 2020. [21 22] This has implications for the clinical guidance for people who have self-harmed, which recommends that assessment by a mental health specialist should follow all episodes of self-harm. [14] Furthermore, such assessments might be particularly challenging in non-face-to-face settings.

Data from NHS Digital [23] showed a 10% decrease in the number of new referrals to NHS mental health services in the six months from 1st April 2020. During the same time period, there was an increase in antidepressant prescribing of around 4% leading to concerns that the increased mental health burden caused by the COVID-19 crisis could be being managed pharmacologically rather than with psychosocial interventions. Our findings suggest this could also have been happening with patients who have harmed themselves. Whilst outpatient and community mental health services adapted to provide alternatives to face-to-face support, our findings suggest that primary care practitioners were less inclined to refer to these services during the early stages of the pandemic. Ongoing work in England to improve community support following self-harm emphasises a need to better align the voluntary sector with primary healthcare services, [24] to ensure that GPs are equipped with the information that they require to make referrals to community self-harm resources as well as NHS services.

Conclusions

Despite the challenges experienced by general practitioners in delivering health care during the three months of the initial wave of COVID-19 in the UK, the management of self-harm was broadly similar to the pre-pandemic comparison period in terms of psychotropic prescribing and GP/nurse consultations. However, the reduced likelihood of referral to

mental health services during March-June 2020 warrants close attention, particularly in the context of increasing prevalence of mental distress in the population and especially so for women. Our findings suggest that COVID-19 may have increased the likelihood that adolescents and young people do not receive psychosocial interventions, with pharmacological intervention alone becoming more likely during the early phase of the crisis. Accessible outpatient and community services that people who have self-harmed can be referred to by their GPs are required.

Author contributions

All authors conceptualised the study and contributed to its design. MJC, LT and SS accessed and verified the data. MJC generated the clinical code lists. MJC and LT managed the data, did statistical analysis. SS drafted the manuscript. All authors critically reviewed the manuscript and approved the final version.

Funding

This work was funded by a UK Research and Innovation COVID-19 Rapid Response Initiative grant (grant reference COV0499), a University of Manchester Presidential Fellowship (awarded to SS) and the NIHR Greater Manchester Patient Safety Translational Research Centre.

Ethics statement

This study was done using data from the CPRD obtained under licence from the UK Medicines and Healthcare products Regulatory Agency (MHRA). The data are provided by patients and collected by the NHS as part of their care and support. All patient data were de-identified; thus, the requirement for patient consent was waived. Individual patients can opt out of sharing their records with the CPRD. The study protocol was approved by the Independent Scientific Advisory Committee (protocol reference number 20_001R2).

Competing interests

NK reports grants and personal fees from the UK Department of Health and Social Care, the National Institute of Health Research (NIHR), the National Institute for Health and Care Excellence (NICE), and the Healthcare Quality and Improvement Partnership, outside the submitted work; works with NHS England on national quality improvement initiatives for suicide and self-harm; is a member of the advisory group for the National Suicide Prevention Strategy of the Department of Health and Social Care; has chaired NICE guideline committees for Self-harm and Depression; and is currently the Topic Advisor for the new NICE Guidelines for self-harm. All other authors declare no competing interests.

For peer review only - http://bmjopen.bmj.com/site/about/guidelines.xhtml

Acknowledgements

We thank Stephen Barlow, Elizabeth Monaghan, Fiona Naylor, and Jonathan Smith (members of the Centre for Mental Health and Safety patient and public involvement and engagement group of the NIHR Greater Manchester Patient Safety Translational Research Centre) for their advisory roles in the study. We would also like to acknowledge all the data providers and general practices that made the anonymised data available for research.

Data sharing

The clinical codes used in this study are available online. The codes are also available from the corresponding author on request. Access to data are available only once approval has been obtained through the individual constituent entities controlling access to the data. The primary care data can be requested via application to the Clinical Practice Research Datalink.

For peer review only

Table 1. Characteristics of cohorts with a primary care-recorded episode of self-harm in the UK			
		Comparison cohort (2010-2019)	COVID-19 cohort (2020)
Total index episodes (between 10th March and 10th June)		48,739	4,238
Gender:			
	Female	29,596 (60.7%)	2,510 (59.2%)
	Male	19,143 (39.3%)	1,728 (40.8%)
Age group (yrs):			
	10-24	20,308 (41.7%)	1,844 (43.5%)
	25-64	25,700 (52.7%)	2,159 (50.9%)
	≥65	2,731 (5.6%)	235 (5.6%)
Practice-level IMD quintile:			
	1 (least deprived)	5,687 (11.7%)	510 (12.0%)
	2	7,003 (14.4%)	600 (14.2%)
	3	8,035 (16.5%)	729 (17.2%)
	4	10,837 (22.2%)	980 (23.1%)
	5 (most deprived)	12,678 (26.0%)	981 (23.2%)
	Unknown	4,499 (9.2%)	438 (10.3%)

For peer review only

For peer review only - http://bmjopen.bmj.com/site/about/guidelines.xhtml

Table 2. Prescriptions of any type of psychotropic medication and any antidepressant drug following a primary care-recorded episode of self-harm in the UK

		Any psychotropic medication prescription			Any antidepressant prescription		
		Comparison Cohort, % (n)	COVID-19 cohort, % (n)	Ratio (95% CI)	Comparison cohort, % (n)	COVID-19 cohort, % (n)	Ratio (95% CI)
All persons		54.0 (26,317)	54.9 (2,328)	1.02 (0.99, 1.05)	47.7 (23,243)	49.4	1.04 (1.00, 1.07)
Gender:							
	Female	53.6 (15,850)	53.9 (1,354)	1.01 (0.97, 1.05)	48.5 (14,338)	49.4	1.02 (0.98, 1.06)
	Male	54.7 (10,467)	56.4 (974)	1.03 (0.99, 1.08)	46.5 (8,905)	49.4	1.06 (1.01, 1.12)
Age group (yrs):							
	10-24	30.9 (6265)	35.1 (648)	1.14 (1.07, 1.22)	26.7 (5,430)	31.5	1.18 (1.10, 1.26)
	25-64	70.8 (18,195)	70.2 (1,515)	0.99 (0.96, 1.02)	63.3 (16,274)	63.8	1.01 (0.97, 1.04)
	≥65	68.0 (1857)	70.2 (165)	1.03 (0.95, 1.13)	56.4 (1,539)	58.7	1.04 (0.93, 1.17)
Practice-level IMD quintile:							
	1 (least deprived)	54.1 (3078)	53.7 (274)	0.99 (0.91, 1.08)	48.4 (2,752)	49.2 (251)	1.02 (0.93, 1.12)
	2	54.0 (3789)	55.0 (330)	1.02 (0.94, 1.10)	47.9 (3,352)	49.5 (297)	1.03 (0.95, 1.13)
	3	54.5 (4378)	56.8 (414)	1.04 (0.98, 1.11)	48.3 (3,883)	50.3 (367)	1.04 (0.97, 1.12)
	4	54.2 (5872)	55.0 (539)	1.02 (0.96, 1.08)	47.5 (5,152)	49.8 (488)	1.05 (0.98, 1.12)
	5 (most deprived)	53.3 (6758)	55.8 (547)	1.05 (0.99, 1.11)	46.9 (5,945)	50.0 (490)	1.07 (1.00, 1.14)
	Unknown	54.3 (2442)	51.1 (224)	0.94 (0.86, 1.04)	48.0 (2,159)	46.1 (202)	0.96 (0.86,1.07)

For peer review only

Table 3. GP/practice nurse consultations following a primary care-recorded episode of self-harm in the UK										
		Face-to-face consultation			Telephone/Video consultation			Face-to-Face or Telephone/Video consultation		
		Comparison cohort, % (n)	COVID-19 cohort, % (n)	Ratio (95% CI)	Comparison cohort, % (n)	COVID-19 cohort, % (n)	Ratio (95% CI)	Comparison cohort, % (n)	COVID-19 cohort, % (n)	Ratio (95% CI)
All persons		80.1 (39,027)	54.3 (2,300)	0.68 (0.66, 0.70)	32.3 (15,723)	67.6 (2,866)	2.10 (2.05, 2.15)	83.2 (40,536)	80.3 (3,403)	0.97 (0.95, 0.98)
Gender:										
	Female	82.5 (24,423)	56.3 (1,412)	0.68 (0.66, 0.71)	33.8 (10,004)	69.8 (1,752)	2.06 (2.00, 2.13)	85.5 (25,300)	82.1 (2,060)	0.96 (0.94, 0.98)
	Male	76.3 (14,604)	51.4 (888)	0.67 (0.64, 0.71)	29.9 (5,719)	64.5 (1,114)	2.16 (2.07, 2.25)	79.6 (15,236)	77.7 (1,343)	0.98 (0.95, 1.00)
Age group (yrs):										
	10-24	74.3 (15,083)	48.2 (888)	0.65 (0.62, 0.68)	24.9 (5,055)	60.5 (1,116)	2.43 (2.33, 2.54)	77.4 (15,720)	74.7 (1,377)	0.96 (0.94, 0.99)
	25-64	84.0 (21,596)	58.0 (1,252)	0.69 (0.67, 0.72)	36.4 (9,341)	72.8 (1,571)	2.00 (1.94, 2.06)	86.9 (22,327)	84.0 (1,813)	0.97 (0.95, 0.99)
	≥65	86.0 (2,348)	68.1 (160)	0.79 (0.72, 0.87)	48.6 (1,327)	76.2 (179)	1.57 (1.45, 1.70)	91.1 (2,489)	90.6 (213)	0.99 (0.95, 1.04)
Practice-level IMD quintile:										
	1 (least deprived)	82.4 (4,687)	56.9 (290)	0.69 (0.64, 0.74)	37.5 (2,135)	68.0 (347)	1.81 (1.69, 1.94)	86.0 (4,891)	81.0 (413)	0.94 (0.90, 0.98)
	2	81.6 (5,711)	50.8 (305)	0.62 (0.58, 0.67)	33.4 (2,340)	70.2 (421)	2.10 (1.97, 2.23)	84.5 (5,918)	80.0 (480)	0.95 (0.91, 0.99)
	3	81.3 (6,534)	55.8 (407)	0.69 (0.64, 0.73)	34.0 (2,731)	66.3 (483)	1.95 (1.84, 2.07)	84.7 (6,807)	80.0 (583)	0.94 (0.91, 0.98)
	4	79.5 (8,613)	53.7 (526)	0.68 (0.64, 0.72)	32.0 (3,472)	67.5 (661)	2.11 (2.00, 2.22)	82.6 (8,949)	80.8 (792)	0.98 (0.95, 1.01)
	5 (most deprived)	79.1 (10,023)	56.2 (551)	0.71 (0.67, 0.75)	29.3 (3,717)	68.3 (670)	2.33 (2.21, 2.45)	81.7 (10,358)	81.9 (803)	1.00 (0.97, 1.03)
	Unknown	76.9 (3,459)	50.5 (221)	0.66 (0.60, 0.72)	29.5 (1,328)	64.8 (284)	2.20 (2.02, 2.39)	80.3 (3,613)	75.8 (332)	0.94 (0.89, 1.00)

For peer review only - http://bmjopen.bmj.com/site/about/guidelines.xhtml

**Table 4. Referrals to mental health services following a primary care-recorded episode of self-harm in Northern Ireland, Scotland and Wales (practices from CPRD GOLD)**			
	Comparison cohort, % (n)	COVID-19 cohort, % (n)	Ratio (95% CI)
All persons	6.5 (780)	3.4 (31)	0.53 (0.37, 0.75)
Gender:			
Female	7.0 (499)	2.4 (12)	0.34 (0.19, 0.60)
Male	5.7 (281)	4.7 (19)	0.83 (0.53, 1.30)
Age group (yrs):			
10-24	7.5 (353)	2.5 (8)	0.33 (0.17, 0.66)
25-64	6.0 (401)	3.6 (19)	0.61 (0.39, 0.96)
≥65	***	***	***

*** Insufficient numbers to present subgroup results

For peer review only

References

1. NICE. The long term care and treatment of self-harm. Clinical Guideline 133.: National Institute of Health and Care Excellence, 2011.
2. Kapur N, Cooper J, O'Connor RC, et al. Non-suicidal self-injury v. attempted suicide: new diagnosis or false dichotomy? *British Journal of Psychiatry* 2013;202(5):326-28. doi: 10.1192/bjp.bp.112.116111
3. Williams R, Jenkins D, Ashcroft D, et al. Diagnosis of physical and mental health conditions in primary care during the COVID-19 emergency: a retrospective cohort study in a deprived UK city. *Lancet Public Health* 2020 23/09/2020.
4. Carr MJ, Steeg S, Webb RT, et al. Effects of the COVID-19 pandemic on primary care-recorded mental illness and self-harm episodes in the UK: a population-based cohort study. *The Lancet Public health* 2021 doi: 10.1016/s2468-2667(20)30288-7
5. Pierce M, Hope H, Ford T, et al. Mental health before and during the COVID-19 pandemic: a longitudinal probability sample survey of the UK population. *The lancet Psychiatry* 2020 doi: 10.1016/s2215-0366(20)30308-4
6. O'Connor RC, Wetherall K, Cleare S, et al. Mental health and wellbeing during the COVID-19 pandemic: longitudinal analyses of adults in the UK COVID-19 Mental Health & Wellbeing study. *The British journal of psychiatry : the journal of mental science* 2020:1-17. doi: 10.1192/bjp.2020.212
7. Centre for Mental Health. How mental health charities are responding to Covid-19 *https://wwwcentreformentalhealthorguk/sites/default/files/publication/download/CentreforMentalHealth_SectorVoicePaperpdf* 2020.
8. Holland KM, Jones C, Vivolo-Kantor AM, et al. Trends in US Emergency Department Visits for Mental Health, Overdose, and Violence Outcomes Before and During the COVID-19 Pandemic. *JAMA psychiatry* 2021 doi: 10.1001/jamapsychiatry.2020.4402
9. John A, DelPozo-Banos M, Gunnell D, et al. Contacts with primary and secondary healthcare prior to suicide: case-control whole-population-based study using person-level linked routine data in Wales, UK, 2000-2017. *The British journal of psychiatry : the journal of mental science* 2020:1-8. doi: 10.1192/bjp.2020.137
10. Carr MJ, Ashcroft DM, Kontopantelis E, et al. Clinical management following self-harm in a UK-wide primary care cohort. *Journal of Affective Disorders* 2016;197:182-88. doi: 10.1016/j.jad.2016.03.013
11. Samaritans. Pushed from pillar to post: Improving the availability and quality of support after self-harm in England. 2020.
12. Mughal F, Troya M, Dikomitis L, et al. Role of the GP in the management of patients with self-harm behaviour: a systematic review. *British Journal of General Practice* 2020;70(694):E364-E73. doi: 10.3399/bjgp20X708257
13. Mroz G, Papoutsi C, Rushforth A, et al. Changing media depictions of remote consulting in COVID-19: analysis of UK newspapers. *British Journal of General Practice* 2021;71(702):E1-E9. doi: 10.3399/bjgp.2020.0967
14. NICE. Self-harm: Longer-term management. Evidence update April 2013. Evidence update 39: National Collaborating Centre for Mental Health, 2013.
15. BBC News. Coronavirus: PM says everyone should avoid office, pubs and travelling 2020 [Available from: https://www.bbc.co.uk/news/uk-51917562 accessed 15.02.2021.
16. Wolf A, Dedman D, Campbell J, et al. Data resource profile: Clinical Practice Research Datalink (CPRD) Aurum. *International Journal of Epidemiology* 2019;48(6):1740-+. doi: 10.1093/ije/dyz034
17. Herrett E, Gallagher AM, Bhaskaran K, et al. Data Resource Profile: Clinical Practice Research Datalink (CPRD). *International Journal of Epidemiology* 2015;44(3):827-36. doi: 10.1093/ije/dyv098

For peer review only - http://bmjopen.bmj.com/site/about/guidelines.xhtml

18. Padmanabhan S, Carty L, Cameron E, et al. Approach to record linkage of primary care data from Clinical Practice Research Datalink to other health-related patient data: overview and implications. *European Journal of Epidemiology* 2019;34(1):91-99. doi: 10.1007/s10654-018-0442-4
19. Ministry of Housing CaLG. English indices of deprivation 2019. 2020 [Available from: https://www.gov.uk/government/statistics/english-indices-of-deprivation-2019.
20. Benchimol EI, Smeeth L, Guttmann A, et al. The REporting of studies Conducted using Observational Routinely-collected health Data (RECORD) Statement. *Plos Medicine* 2015;12(10) doi: 10.1371/journal.pmed.1001885
21. Kooth. DATA RELEASE JULY 06 2020. Week 16: How Covid-19 is Affecting The Mental Health of Children and Young People (CYP). 2020 [Available from: https://xenzone.com/wp-content/uploads/2020/07/Week16CYPv2.pdf.
22. Samaritans. **Coronavirus, young people and self-harm** 2020 [Available from: https://www.samaritans.org/about-samaritans/research-policy/understanding-our-callers-during-covid-19-pandemic/coronavirus-young-people-and-self-harm/.
23. Armitage R. Antidepressants, primary care, and adult mental health services in England during COVID-19. *The lancet Psychiatry* 2021;8(2):e3-e3. doi: 10.1016/s2215-0366(20)30530-7
24. National Collaborating Central for Mental Health. The Community Mental Health Framework for Adults and Older Adults. *https://wwwenglandnhsuk/wp-content/uploads/2019/09/community-mental-health-framework-for-adults-and-older-adultspdf* 2019.

For peer review only - http://bmjopen.bmj.com/site/about/guidelines.xhtml

Supplementary material

Table S1: The RECORD statement – checklist of items, extended from the STROBE statement, that should be reported in observational studies using routinely collected health data.

	Item No.	STROBE items	Location in manuscript where items are reported	RECORD items	Location in manuscript where items are reported
**Title and abstract**					
	1	(a) Indicate the study's design with a commonly used term in the title or the abstract (b) Provide in the abstract an informative and balanced summary of what was done and what was found		RECORD 1.1: The type of data used should be specified in the title or abstract. When possible, the name of the databases used should be included.<br><br>RECORD 1.2: If applicable, the geographic region and timeframe within which the study took place should be reported in the title or abstract.<br><br>RECORD 1.3: If linkage between databases was conducted for the study, this should be clearly stated in the title or abstract.	Abstract: 'Design and setting' (page 2)<br><br>Abstract: 'Method', (page 2)
**Introduction**					
Background rationale	2	Explain the scientific background and rationale for the investigation being reported			Introduction: paragraphs 1-5 (pages 4-5)
Objectives	3	State specific objectives, including any prespecified hypotheses			Introduction: paragraph 5 (page 5)
**Methods**					
Study Design	4	Present key elements of study design early in the paper			Methods: '*Study design, data sources, and participants'* (page 6)
Setting	5	Describe the setting, locations, and relevant dates, including periods of			Methods: '*Study design, data*

For peer review only

		recruitment, exposure, follow-up, and data collection			*sources, and participants'* (page 6)
Participants	6	*(a) Cohort study* - Give the eligibility criteria, and the sources and methods of selection of participants. Describe methods of follow-up<br>*Case-control study* - Give the eligibility criteria, and the sources and methods of case ascertainment and control selection. Give the rationale for the choice of cases and controls<br>*Cross-sectional study* - Give the eligibility criteria, and the sources and methods of selection of participants<br><br>*(b) Cohort study* - For matched studies, give matching criteria and number of exposed and unexposed<br>*Case-control study* - For matched studies, give matching criteria and the number of controls per case		RECORD 6.1: The methods of study population selection (such as codes or algorithms used to identify subjects) should be listed in detail. If this is not possible, an explanation should be provided.<br><br>RECORD 6.2: Any validation studies of the codes or algorithms used to select the population should be referenced. If validation was conducted for this study and not published elsewhere, detailed methods and results should be provided.<br><br>RECORD 6.3: If the study involved linkage of databases, consider use of a flow diagram or other graphical display to demonstrate the data linkage process, including the number of individuals with linked data at each stage.	Methods: '*Study design, data sources, and participants'* (paragraph 3) (page 6) and '*Exposures, outcomes and covariates'* (page 7)<br><br>Results: paragraph 1 (page 8)
Variables	7	Clearly define all outcomes, exposures, predictors, potential confounders, and effect modifiers. Give diagnostic criteria, if applicable.		RECORD 7.1: A complete list of codes and algorithms used to classify exposures, outcomes, confounders, and effect modifiers should be provided. If these cannot be reported, an explanation should be provided.	Methods: '*Exposures, outcomes and covariates'* (page 7)
Data sources/ measurement	8	For each variable of interest, give sources of data and details of methods of assessment (measurement).<br>Describe comparability of assessment methods if there is more than one group			Methods: '*Exposures, outcomes and covariates'* (page 7)

For peer review only - http://bmjopen.bmj.com/site/about/guidelines.xhtml

Bias	9	Describe any efforts to address potential sources of bias			Methods: 'Analysis' (page 7)
Study size	10	Explain how the study size was arrived at			Methods: '*Study design, data sources, and participants'* (paragraph 2) (page 6)
Quantitative variables	11	Explain how quantitative variables were handled in the analyses. If applicable, describe which groupings were chosen, and why			Methods: '*Study design, data sources, and participants'* (paragraph 2) (page 6)'
Statistical methods	12	(a) Describe all statistical methods, including those used to control for confounding<br>(b) Describe any methods used to examine subgroups and interactions<br>(c) Explain how missing data were addressed<br>(d) *Cohort study* - If applicable, explain how loss to follow-up was addressed<br>*Case-control study* - If applicable, explain how matching of cases and controls was addressed<br>*Cross-sectional study* - If applicable, describe analytical methods taking account of sampling strategy<br>(e) Describe any sensitivity analyses			Methods: 'Analysis' (page 7)
Data access and cleaning methods		..		RECORD 12.1: Authors should describe the extent to which the investigators had access to the database population used to create the study population.	'Author contributions', (page 12)

For peer review only - http://bmjopen.bmj.com/site/about/guidelines.xhtml

				RECORD 12.2: Authors should provide information on the data cleaning methods used in the study.	
Linkage		..		RECORD 12.3: State whether the study included person-level, institutional-level, or other data linkage across two or more databases. The methods of linkage and methods of linkage quality evaluation should be provided.	Methods: '*Study design, data sources, and participants'* (page 6)
**Results**					
Participants	13	(a) Report the numbers of individuals at each stage of the study (*e.g.*, numbers potentially eligible, examined for eligibility, confirmed eligible, included in the study, completing follow-up, and analysed)<br>(b) Give reasons for non-participation at each stage.<br>(c) Consider use of a flow diagram		RECORD 13.1: Describe in detail the selection of the persons included in the study (*i.e.,* study population selection) including filtering based on data quality, data availability and linkage. The selection of included persons can be described in the text and/or by means of the study flow diagram.	Results: paragraph 1 (page 8)
Descriptive data	14	(a) Give characteristics of study participants (*e.g.*, demographic, clinical, social) and information on exposures and potential confounders<br>(b) Indicate the number of participants with missing data for each variable of interest<br>(c) *Cohort study* - summarise follow-up time (*e.g.*, average and total amount)			Results: paragraph 1 (page 8)<br><br>Methods: '*Study design, data sources, and participants'* (paragraph 2) (page 6)<br><br>Methods: '*Study design, data sources, and participants'* (paragraph 2) (page 6)

For peer review only - http://bmjopen.bmj.com/site/about/guidelines.xhtml

Outcome data	15	*Cohort study* - Report numbers of outcome events or summary measures over time<br>*Case-control study* - Report numbers in each exposure category, or summary measures of exposure<br>*Cross-sectional study* - Report numbers of outcome events or summary measures			Table 1 (page 13)
Main results	16	(a) Give unadjusted estimates and, if applicable, confounder-adjusted estimates and their precision (e.g., 95% confidence interval). Make clear which confounders were adjusted for and why they were included<br>(b) Report category boundaries when continuous variables were categorized<br>(c) If relevant, consider translating estimates of relative risk into absolute risk for a meaningful time period			Results (page 8) and Tables 1-4 (Pages 13-16)
Other analyses	17	Report other analyses done—e.g., analyses of subgroups and interactions, and sensitivity analyses			
**Discussion**					
Key results	18	Summarise key results with reference to study objectives			Discussion: 'Summary' (Page 9)
Limitations	19	Discuss limitations of the study, taking into account sources of potential bias or imprecision. Discuss both direction and magnitude of any potential bias		RECORD 19.1: Discuss the implications of using data that were not created or collected to answer the specific research question(s). Include discussion of misclassification bias, unmeasured confounding, missing data, and changing	Discussion: 'Strengths and limitations' (page 9)

For peer review only - http://bmjopen.bmj.com/site/about/guidelines.xhtml

				eligibility over time, as they pertain to the study being reported.	
Interpretation	20	Give a cautious overall interpretation of results considering objectives, limitations, multiplicity of analyses, results from similar studies, and other relevant evidence			Discussion: 'Implications for research and practice' (page 10)
Generalisability	21	Discuss the generalisability (external validity) of the study results			Discussion: 'Strengths and limitations' (page 9)
**Other Information**					
Funding	22	Give the source of funding and the role of the funders for the present study and, if applicable, for the original study on which the present article is based			Funding statement (page 11)
Accessibility of protocol, raw data, and programming code		..		RECORD 22.1: Authors should provide information on how to access any supplemental information such as the study protocol, raw data, or programming code.	Data sharing statement (Page 12)

*Reference: Benchimol EI, Smeeth L, Guttmann A, Harron K, Moher D, Petersen I, Sørensen HT, von Elm E, Langan SM, the RECORD Working Committee. The REporting of studies Conducted using Observational Routinely-collected health Data (RECORD) Statement. *PLoS Medicine* 2015; in press.

*Checklist is protected under Creative Commons Attribution (CC BY) license.

For peer review only

**Table S2. Prescriptions of specific psychotropic medication types following a primary care-recorded episode of self-harm in the UK**			
	Comparison cohort, % (n)	COVID-19 cohort, % (n)	Ratio (95% CI)
Antidepressants:	47.7 (23,243)	49.4 (2,095)	1.04 (1.00, 1.07)
Antipsychotics:	10.8 (5,285)	12.3 (521)	1.13 (1.04, 1.23)
Anxiolytics/hypnotics:	17.6 (8,577)	15.5 (657)	0.88 (0.82, 0.95)
Mood stabilisers:	5.7 (2,763)	5.6 (238)	0.99 (0.87, 1.13)
Stimulants:	0.8 (369)	1.0 (44)	1.37 (1.00, 1.87)

For peer review only

BMJ Open

Primary care clinical management following self-harm during the first wave of COVID-19 in the UK: population-based cohort study

Journal:	*BMJ Open*
Manuscript ID	bmjopen-2021-052613.R1
Article Type:	Original research
Date Submitted by the Author:	30-Nov-2021
Complete List of Authors:	Steeg, Sarah; The University of Manchester Faculty of Biology Medicine and Health<br>Carr, M; The University of Manchester Faculty of Biology Medicine and Health<br>Trefan, Laszlo; The University of Manchester Faculty of Biology Medicine and Health, Centre for Mental Health and Safety, University of Manchester, Manchester, UK. Manchester Academic Health Science Centre, University of Manchester, Manchester, UK<br>Ashcroft, Darren; University of Manchester<br>Kapur, Navneet; University of Manchester, Centre for suicide prevention<br>Nielsen, Emma; University of Nottingham<br>McMillan, Brian; The University of Manchester, Centre for Primary Care and Health Services Research;<br>Webb, Roger; The University of Manchester, School of Community Based Medicine
**Primary Subject Heading**:	Mental health
Secondary Subject Heading:	Epidemiology, General practice / Family practice, Health services research
Keywords:	PRIMARY CARE, Suicide & self-harm < PSYCHIATRY, PUBLIC HEALTH, COVID-19

SCHOLARONE™
Manuscripts

I, the Submitting Author has the right to grant and does grant on behalf of all authors of the Work (as defined in the below author licence), an exclusive licence and/or a non-exclusive licence for contributions from authors who are: i) UK Crown employees; ii) where BMJ has agreed a CC-BY licence shall apply, and/or iii) in accordance with the terms applicable for US Federal Government officers or employees acting as part of their official duties; on a worldwide, perpetual, irrevocable, royalty-free basis to BMJ Publishing Group Ltd ("BMJ") its licensees and where the relevant Journal is co-owned by BMJ to the co-owners of the Journal, to publish the Work in this journal and any other BMJ products and to exploit all rights, as set out in our licence.

The Submitting Author accepts and understands that any supply made under these terms is made by BMJ to the Submitting Author unless you are acting as an employee on behalf of your employer or a postgraduate student of an affiliated institution which is paying any applicable article publishing charge ("APC") for Open Access articles. Where the Submitting Author wishes to make the Work available on an Open Access basis (and intends to pay the relevant APC), the terms of reuse of such Open Access shall be governed by a Creative Commons licence – details of these licences and which Creative Commons licence will apply to this Work are set out in our licence referred to above.

Other than as permitted in any relevant BMJ Author's Self Archiving Policies, I confirm this Work has not been accepted for publication elsewhere, is not being considered for publication elsewhere and does not duplicate material already published. I confirm all authors consent to publication of this Work and authorise the granting of this licence.

For peer review only

Primary care clinical management following self-harm during the first wave of COVID-19 in the UK: population-based cohort study

Sarah Steeg*[1,2], Matthew J Carr*[1, 2-4], Laszlo Trefan [1,2], Darren M Ashcroft [1, 2-4], Navneet Kapur [1,2,4,5], Emma Nielsen [6], Brian McMillan [7], Roger T Webb [1,2,4].

*Joint first authors

Sarah Steeg, PhD, Presidential Research Fellow

Matthew J Carr, PhD, Research Fellow

Laszlo Trefan, PhD, Research Fellow

Darren M Ashcroft, PhD FRPharmS, Professor of Pharmacoepidemiology

Navneet Kapur, FRCPsych, MD, Professor of Psychiatry and Population Health

Emma Nielsen, PhD, Research Fellow

Brian McMillan, PhD, Clinical Lecturer

Roger T Webb, PhD, Professor of Mental Health Epidemiology

[1] Centre for Mental Health and Safety, University of Manchester, Manchester, UK.

[2] Manchester Academic Health Science Centre, University of Manchester, Manchester, UK.

[3] Centre for Pharmacoepidemiology and Drug Safety, National Institute for Health Research Greater Manchester Patient Safety Translational Research Centre, University of Manchester, Manchester, UK.

[4] National Institute for Health Research Greater Manchester Patient Safety Translational Research Centre, University of Manchester, Manchester, UK.

[5] Greater Manchester Mental Health NHS Foundation Trust, Manchester, UK.

[6] Self-harm Research Group, School of Psychology, University of Nottingham, UK.

[7] Centre for Primary Care and Health Services Research, University of Manchester, Manchester, UK.

Corresponding author: Sarah Steeg, sarah.steeg@manchester.ac.uk; 2.308 Jean McFarlane Building, University of Manchester, Oxford Road, Manchester, M13 9PL, UK; Tel +44(0)161 275 0718, Fax: +44 (0)161 275 0716

For peer review only - http://bmjopen.bmj.com/site/about/guidelines.xhtml

Abstract

Objectives

A substantial reduction in self-harm recorded in primary care occurred during the first wave of COVID-19 but effects on primary care management of self-harm are unknown. Our objectives were to examine the impact of COVID-19 on clinical management within three months of an episode of self-harm.

Design

Retrospective cohort study.

Setting

UK primary care.

Participants

4,238 patients with an index episode of self-harm recorded in UK primary care during the COVID-19 first-wave period (10th March-10th June 2020) compared to 48,739 patients in a pre-pandemic comparison period (10th March-10th June, 2010-2019).

Outcome measures

Using data from the UK Clinical Practice Research Datalink we compared cohorts of patients with an index self-harm episode recorded during the pre-pandemic period versus the COVID-19 first-wave period. Patients were followed up for three months to capture subsequent GP/practice nurse consultation, referral to mental health services and psychotropic medication prescribing. We examined differences by gender, age group and Index of Multiple Deprivation (IMD) quintile.

Results

Likelihood of having at least one GP/practice nurse consultation was broadly similar (83.2% vs. 80.3% in the COVID-19 cohort). The proportion of patients referred to mental health services in the COVID-19 cohort (4.2%) was around two-thirds of that in the pre-pandemic cohort (6.1%). Similar proportions were prescribed psychotropic medication within 3 months in the pre-pandemic (54.0%) and COVID-19 first-wave (54.9%) cohorts.

Conclusions

For peer review only - http://bmjopen.bmj.com/site/about/guidelines.xhtml

For peer review only

Despite the challenges experienced by primary healthcare teams during the initial COVID-19 wave, prescribing and consultation patterns following self-harm were broadly similar to pre-pandemic levels. We found no evidence of widening of digital exclusion in terms of access to remote consultations. However, the reduced likelihood of referral to mental health services warrants attention. Accessible outpatient and community services for people who have self-harmed are required as the COVID-19 crisis recedes and the population faces new challenges to mental health.

Keywords: general practice; self-harm; mental health; COVID-19; psychotropic drugs; epidemiology.

Strengths and limitations of this study

- First study examining effects of Covid-19 on primary care management following self-harm in the UK
- Findings are based on a large number of general practices across the UK, using data from the Clinical Practice Research Datalink
- Data broadly representative of geographical coverage, area-level deprivation, age, and sex in England
- Data accuracy is determined by the quality of the information inputted by contributing practices.

For peer review only

Introduction

Prompt clinical intervention and follow-up is recommended for people who have recently self-harmed, in part due to their increased risks of suicide. [1] Self-harm includes intentional self-poisoning and self-injury and can involve varying degrees of suicidal intent. [2] All episodes of self-harm should be followed by comprehensive mental health assessment to identify psychosocial needs and address risks of further self-harm and suicide. [1] This has become more challenging due to the disruption to UK health services caused by the COVID-19 pandemic. Fluctuations in infection rates, lockdown restrictions and public health messaging has had a significant impact on the numbers of people accessing primary care services, with considerable reductions in presentation rates found for a number of physical and mental health conditions, [3] including self-harm. [4] The pandemic and its pervasive impacts on everyday life has also had a detrimental impact on the mental health of the population [5] and the first wave of COVID-19 and lockdown may have led to an increase in the prevalence of suicidal ideation. [6] This unique combination of factors has led to a shortfall in the numbers of people receiving support from healthcare services after harming themselves.

In April 2020, during the first UK COVID-19 lockdown, rates of primary care-recorded incident self-harm in the UK were 38% lower than expected based on trends that occurred during the previous ten years. [4] While rates of help seeking gradually returned towards expected levels through the subsequent months up to September 2020, further regional COVID-19 containment restrictions and national lockdowns from autumn 2020 into winter 2021 are likely to have additionally affected rates of help seeking. Some people sought help from alternative sources whilst the UK was in its first lockdown; for example, some mental health charities reported increases in demand for services such as helplines. [7] One study in the US reported emergency hospital presentations following suicide attempts had increased to higher than pre-pandemic levels following an initial reduction, [8] suggesting that observed reductions in help seeking in primary care did not necessarily reflect population need. Furthermore, a living systematic review on the impacts of COVID-19 on suicidal behaviour globally found that while the majority of studies reported a decrease in health service contacts for self-harm, some identified an increased likelihood of using more lethal methods. [9]

Primary care settings provide vital support for people who have self-harmed. A recent study found that 26% of people sought help from their general practitioner (GP) in the week prior to suicide, with self-harm a common reason for contact.[10] Previous research found that 15% of patients with an episode of self-harm recorded in primary care were referred to mental health services from their GP within a year. [11] A recent report found that, among people referred to mental health services, their

For peer review only - http://bmjopen.bmj.com/site/about/guidelines.xhtml

GP was the most common referral route. [12] However, this report highlighted many barriers that exist for people accessing support following self-harm. [12] GPs, as well as patients, have reported struggling to find appropriate self-harm services, with limited referral options and shortages in community services identified. [13]

It is unknown how clinical management following self-harm has been affected by the COVID-19 pandemic. Although GPs continued to offer consultations to patients throughout the first wave in the spring of 2020, with most consultations taking place remotely, [14] recently conducted research found lower rates of GP referral to mental health services following primary care-recorded common mental illnesses and self-harm episodes. [4] There has also been concern that the pandemic could contribute further to digital exclusion; patients living with greater levels of socioeconomic deprivation being further excluded from remote clinical care. [15] The degree to which primary care management of people who had self-harmed was impacted during the first COVID-19 wave is unknown. This is an important research question, because self-harm is a key risk factor for suicide and requires specific timely intervention. [16]

In the UK, a nationwide lockdown was imposed on 23rd March 2020, with public health messaging to encourage people to avoid contact with others announced the week before this. [17] We aimed to examine clinical management of self-harm during the three months following the beginning of the UK COVID-19 containment measures and national lockdown, using data from a pre-pandemic comparison period to examine effects of COVID-19. Our specific objectives were to:

(i) Identify two cohorts of patients presenting with an index episode of self-harm, comprising pre-pandemic versus COVID-19 first-wave time periods.

(ii) Estimate the probabilities (%) of receiving a new psychotropic medication prescription (by drug type), frequency of subsequent GP or practice nurse consultations, and referral to mental health services within 3 months of the index self-harm episode

(iii) Estimate ratios comparing probabilities of psychotropic medication prescribing, referral to mental health services and GP or practice nurse consultation (by face-to-face and telephone) within three months of the index episode, between the COVID-19 and pre-pandemic comparison cohorts.

For peer review only - http://bmjopen.bmj.com/site/about/guidelines.xhtml

Methods

Study design, data sources, and participants

We conducted a cohort study using anonymised primary care data from the Clinical Practice Research Datalink (CPRD) Aurum and GOLD databases. [18 19] Both Aurum and GOLD contain data extracted from electronic patient record platforms; specifically, EMIS® and Vision®. These platforms are used to record information about patients including content of patient consultations, signs and symptoms, diagnoses, tests, medication prescriptions and referrals. The Aurum database includes general practices based in England that contribute data using the EMIS® clinical system. The GOLD database is extracted from the Vision® system, with most of its contributing general practices based in Northern Ireland, Scotland and Wales. The Aurum database is broadly representative of geographical coverage, area-level deprivation, age, and sex distributions of the population of England, [18] whist GOLD is broadly representative of the sociodemographic profile of the whole UK population. [19] To avoid including duplicate general practices in the Aurum and GOLD databases, we excluded English practices in our analyses of GOLD data. The CPRD includes information on patient demographics, consultations, symptoms, diagnoses, medication prescriptions and referrals to secondary care. We also obtained information on Index of Multiple Deprivation (IMD) score linked at general practice level. [20] The IMD is a single score derived from seven domains of areas-based deprivation measures: income, employment, education, health, crime, barriers to housing and services, and living environment.. [21] The IMD is a relative measure of deprivation between areas. The postcode of the general practice was linked to the IMD score of its corresponding lower super output area in England and Wales (an area typically containing around 1500 residents), super output area in Northern Ireland (containing an average of 2100 individuals) or datazone in Scotland (containing a population of between 500 and 1000).

Analysis was conducted on pooled Aurum and GOLD data. We compared two cohorts of patients: (i) those with an index self-harm episode recorded in a pre-pandemic comparison period (between 10th March to 10th June, 2010-2019) and those with an index self-harm episode recorded in the COVID-19 first-wave period (10th March to 10th June 2020). Patients in each cohort were followed up for three months to capture psychotropic medication prescribing, referral to mental health services and GP or practice nurse consultations. To be included in either of the two study cohorts, patients must have been aged 10 years or older and registered with a contributing practice, deemed by the CPRD as providing up-to-standard data, for at least one year prior to the date of the index self-harm episode. Patients with less than 3 months of follow-up time in the CPRD were excluded from our analyses. The cohorts were restricted to patients with records that were deemed acceptable by the CPRD for research purposes, which excluded patients with missing data on sex or age. IMD data were missing for 9.2% of the pre-pandemic comparison cohort and 10.3% of the COVID-19 first-wave cohort (Table 1). In terms of missing outcome data, if there was no record of psychotropic medication prescribing, face-to-face or remote consultation and referral to mental health service, the outcome was not recorded as present.

For peer review only - http://bmjopen.bmj.com/site/about/guidelines.xhtml
For peer review

The study was approved by the Independent Scientific Advisory Committee (protocol number 20_001RA2).

Exposures, outcomes and covariates

Index episodes of self-harm were identified from Read, SNOMED and EMIS codes: SNOMED CT [22] is a clinical vocabulary that is readable by computers. Used internationally, it is the recommended structured clinical vocabulary to record electronic patient information in the National Health Service in England. EMIS and Read codes are further coding systems used to capture clinical terms used in patient records. [23] Codes relating to intentional self-poisoning and self-injury episodes, of varying degrees of suicidal intent, were included. To identify individuals' first record of self-harm, we applied a retrospective analysis period during which a patient was required to have been registered at the practice for at least 1 year before an incident episode. We examined any psychotropic medication and specific psychotropic medication types including antidepressants, antipsychotics, anxiolytic/hypnotics, mood stabilisers and stimulants (https://clinicalcodes.rss.mhs.man.ac.uk/medcodes/article/173/). Mode of consultation was grouped into face-to-face and video/telephone consultations, with a GP or practice nurse. Information on referral to mental health services were identified using two CPRD fields: a 'psychiatry' code in the Family Health Services Authority (FHSA) speciality variable and codes of 'mental illness', 'child and adolescent psychiatry', 'forensic psychiatry', 'psychotherapy', 'old age psychiatry', 'clinical psychology', 'adult psychiatry' and 'community psychiatric nurse' in the National Health Service (NHS) specialty field. [11] We combined information from both the FHSA and NHS specialty fields to construct a binary specialist mental health services referral indicator. All code lists were verified by senior clinical academics as part of a previous study [4] and are available online (https://clinicalcodes.rss.mhs.man.ac.uk/medcodes/article/173/). We examined frequencies and rate ratios between the pre-pandemic and COVID-19 first-wave cohorts by the following covariates: gender, age group (10-24, 25-64 and 65 and older) and practice-level IMD quintile. Subgroup categories were derived to avoid reporting cell counts less than 10; if cell counts were found to be less than 10, subgroups were collapsed.

Analysis

Frequencies and probabilities of GP/nurse consultation, referral to mental health services and psychotropic medication prescribing during the antecedent period were estimated and compared to observed values during the COVID-19 first-wave period. The modelling was conducted using modified Poisson regression in a generalised linear modelling framework with a log-link function and a robust variance estimator with analyses stratified by gender, age group and practice-level IMD quintile. This study was conducted in accordance with REporting of studies Conducted using Observational Routinely-collected health Data guidance (RECORD; Table S1). [24]

For peer review only - http://bmjopen.bmj.com/site/about/guidelines.xhtml

Patient and public involvement

A panel of four service users and carers with lived experience of health services following self-harm collaborated with the research team to plan the study and interpret results. Panel members reviewed findings based on their experiences of health services for self-harm and the COVID-19 pandemic and associated societal restrictions. Over two workshops, panel members met with the corresponding author of the study to review the results of the study and provide feedback on their visual presentation. The group is linked with the National Institute Health Research Greater Manchester Patient Safety Translational Research Centre (NIHR GM PSTRC).

Results

A total of 48,739 patients had an index episode of self-harm recorded in the UK during the pre-pandemic comparison period and 4,238 were recorded in the COVID-19 first-wave cohort (Table 1). The gender, age and deprivation profiles of the two cohorts were broadly similar, with the majority of recorded self-harm episodes by women and more self-harm episodes recorded in practices in areas of higher deprivation.

Unsurprisingly, the likelihood of patients receiving a remote GP/practice nurse consultation within 3 months of a self-harm episode was higher in the COVID-19 'first-wave' cohort (67.7%) than in the pre-pandemic comparison cohort (32.3%, ratio 2.10, CI 2.05 to 2.15) (Table 2). Although the overall likelihood of having a GP/practice nurse consultation was slightly lower in the COVID-19 cohort (80.3%) than in the pre-pandemic comparison cohort (83.2%), ratio 0.97, CI 0.96 to 0.98, this pattern did not apply to all demographic groups. Men, patients aged 65 years and over and those registered with practices in the two most deprived quintiles were equally likely to have had a GP/practice nurse consultation in the pre-pandemic and COVID-19 first-wave cohorts. With respect to remote consultation specifically, there was no difference between practice deprivation-level and likelihood of receiving this form of management.

Overall, 4.2% of patients (179/4238) were referred to mental health services in the COVID-19 first-wave cohort, a significant reduction versus the probability observed in the pre-pandemic comparison cohort (6.1%, ratio 0.70, CI 0.60 to 0.81) (Table 3). The reduction in likelihood of being referred to mental health servicers was not observed for patients aged 65 years and over (ratio 1.66, CI 0.98 to 2.80), p value for effect modification by age group = 0.01).

Just over half of patients in the pre-pandemic (54.0%) and COVID-19 first-wave (54.9%) cohorts were prescribed psychotropic medication within three months of the index self-harm episode (Table 4). The likelihoods of receiving such treatment were broadly similar across gender and deprivation quintiles. Among patients aged 10-24 years, those in the

For peer review only - http://bmjopen.bmj.com/site/about/guidelines.xhtml

COVID-19 first-wave cohort were more likely to be prescribed psychotropic medication (ratio 1.14, 95% CI 1.07 to 1.22). Considering prescriptions for antidepressant medication specifically, probabilities were broadly similar in the pre-pandemic and COVID-19 first-wave cohorts, though they were higher among young people aged 10-24 years in the COVID-19 first-wave cohort (ratio 1.18, CI 1.10 to 1.26). Details on prescribing of antipsychotic, anxiolytic/hypnotic, mood stabilisers and stimulants are in Table 5.

Discussion

Summary

Pre-pandemic and COVID-19 first-wave cohorts of patients with an index episode of self-harm recorded in primary care had similar gender, age and deprivation profiles. Similar proportions were prescribed psychotropic medication within three months of their index self-harm episode - just over half in both the pre-pandemic and COVID-19 first-wave cohorts. However, patients aged 10-24 years in the COVID-19 first-wave cohort were more likely to be prescribed psychotropic medication than in the preceding years. Overall, the likelihood of having at least one GP/practice nurse consultation was slightly lower in the COVID-19 first-wave cohort, although there was no such difference observed among men, patients aged 65 years and over, and those registered with practices located in more deprived areas. Patients in more deprived practice populations who had harmed themselves were just as likely to consult face-to-face or remotely with a GP or a practice nurse than those in less deprived populations. The proportion of patients referred to mental health services in the COVID-19 first-wave cohort was around two-thirds of that in the pre-pandemic comparison cohort.

Strengths and limitations

The main strength of our study is the broadly representative data source that included a large number of general practices across the UK. CPRD Aurum is broadly representative of geographical coverage, area-level deprivation, age, and sex in England [18] while CPRD GOLD dataset is broadly representative of the UK age and sex profile.[19] This enables us to make inferences at national level about how the pandemic affected primary care clinical management of patients who have self-harmed. However, our findings may not be generalisable to countries experiencing different degrees of COVID-19 containment measures and societal restrictions and those with much lower levels of access to universal healthcare. There are some limitations in utilising anonymised primary care records. The data are extracted from GP information systems and their accuracy is determined by the quality of the information inputted by contributing practices. The rapid adaptations to working methods that were necessary during the early stages of the pandemic may have affected accuracy of clinical coding. Some of the self-harm episodes recorded in primary care would have been emergency department presentations that were subsequently added to the patient's primary care record. Suicidal intent

For peer review only - http://bmjopen.bmj.com/site/about/guidelines.xhtml

specific to each self-harm episode could not be examined in this study. We were also unable to examine clinical management outcomes with no corresponding referral code, such as patients being advised to self-refer to third sector organisations or Improving Access to Psychological Therapies services. Future research using linked Hospital Episode Statistics will enable separate examination of emergency department self-harm presentations. Finally, we were unable to examine suicide deaths and other causes of mortality in this study due to unavailability of linked mortality records at the time of analysis.

Comparison with existing literature

Previous research found that reductions in help-seeking were greatest among patients registered at practices located in more deprived areas.[4] Our study found that patients at practices in the two most deprived quintiles who did seek help had similar rates of psychotropic medication prescribing as those at practices in less deprived areas, in both the pre-pandemic comparison and COVID-19 first-wave cohorts. Similarly, while there was a clear deprivation gradient in likelihood of remote consultation for self-harm prior to the pandemic, with those in areas of lower deprivation less likely to have remote consultation, the abrupt switch to remote consultations once the COVID-19 crisis had commenced did not lead to widening of existing inequalities in this respect. Evidence conducted prior to the COVID-19 pandemic indicated there was potential for the growth in virtual consultations to widen existing disparities in access to health. [25] Our findings suggest that for people who did seek help, there was no exacerbation of existing inequalities due to COVID-19 in these particular aspects of primary care clinical management, despite the growth in remote consultation numbers. In other words, we found no evidence of digital exclusion from self-harm care during the pandemic due to socioeconomic influences.

Likelihood of referral to NHS mental health services was lower during the first wave of COVID-19 than during the pre-pandemic comparison period, particularly for patients aged under 65 years. Younger people have been found to have been particularly negatively affected by the pandemic. Individuals aged 18-29 years reported greater increases in suicidal ideation over the first six weeks of the UK's lockdown than other groups, [6] and were found to have the greatest deteriorations in mental health. [5] Furthermore, working age adults were previously identified as having the greatest reductions in help seeking for mental illness and self-harm during April 2020.[4]

Implications for research and practice

Evidence shows the number of people seeking mental health help, including for self-harm, from non-NHS services such as via digital platforms and helplines increased during the second quarter of 2020. [26 27] This has implications for the clinical guidance for people who have self-harmed, which recommends that assessment by a mental health specialist should follow all episodes of self-harm. [16] Furthermore, such assessments might be particularly challenging in non-face-to-face settings.

For peer review only - http://bmjopen.bmj.com/site/about/guidelines.xhtml

Data from NHS Digital [28] showed a 10% decrease in the number of new referrals to NHS mental health services in the six months from 1st April 2020. During the same time period, there was an increase in antidepressant prescribing of around 4% leading to concerns that the increased mental health burden caused by the COVID-19 crisis could be being managed pharmacologically rather than with psychosocial interventions. Our findings suggest this could also have been happening with patients who have harmed themselves. Whilst outpatient and community mental health services adapted to provide alternatives to face-to-face support, our findings suggest that primary care practitioners were less inclined to refer to these services during the early stages of the pandemic. Ongoing work in England to improve community support following self-harm emphasises a need to better align the voluntary sector with primary healthcare services, [29] to ensure that GPs are equipped with the information that they require to make referrals to community self-harm resources as well as NHS services.

Conclusions

Despite the challenges experienced by general practitioners in delivering health care during the three months of the initial wave of COVID-19 in the UK, the management of self-harm was broadly similar to the pre-pandemic comparison period in terms of psychotropic prescribing and GP/nurse consultations. We found no evidence of digital exclusion due to socioeconomic influences during the pandemic, in terms of likelihood of remote consultation. However, the reduced likelihood of referral to mental health services during March-June 2020 warrants close attention, particularly in the context of increasing prevalence of mental distress in the population. Our findings suggest that COVID-19 may have increased the likelihood that adolescents and young people do not receive psychosocial interventions, with pharmacological intervention alone becoming more likely during the early phase of the crisis. Accessible outpatient and community services that people who have self-harmed can be referred to by their GPs are required.

Author contributions

SS, MJC, LT, DMA, NK, EN, BM and RTW conceptualised the study and contributed to its design. MJC, LT and SS acquired, accessed and verified the data. MJC generated the clinical code lists. MJC and LT managed the data and conducted the statistical analysis. SS, MJC, LT, DMA, NK, EN, BM and RTW interpreted the results. SS drafted the manuscript. SS, MJC, LT, DMA, NK, EN, BM and RTW critically reviewed the manuscript and approved the final version. SS accepts full responsibility for the finished work and the conduct of the study, had access to the data, and controlled the decision to publish.

Funding

For peer review only - http://bmjopen.bmj.com/site/about/guidelines.xhtml

This work was funded by a UK Research and Innovation COVID-19 Rapid Response Initiative grant (grant reference COV0499), a University of Manchester Presidential Fellowship (awarded to SS) and the NIHR Greater Manchester Patient Safety Translational Research Centre.

Ethics statement

This study was done using data from the CPRD obtained under licence from the UK Medicines and Healthcare products Regulatory Agency (MHRA). The data are provided by patients and collected by the NHS as part of their care and support. All patient data were de-identified; thus, the requirement for patient consent was waived. Individual patients can opt out of sharing their records with the CPRD. The study protocol was approved by the Independent Scientific Advisory Committee (protocol reference number 20_001R2).

Competing interests

NK reports grants and personal fees from the UK Department of Health and Social Care, the National Institute of Health Research (NIHR), the National Institute for Health and Care Excellence (NICE), and the Healthcare Quality and Improvement Partnership, outside the submitted work; works with NHS England on national quality improvement initiatives for suicide and self-harm; is a member of the advisory group for the National Suicide Prevention Strategy of the Department of Health and Social Care; has chaired NICE guideline committees for Self-harm and Depression; and is currently the Topic Advisor for the new NICE Guidelines for self-harm. All other authors declare no competing interests.

Acknowledgements

We thank Stephen Barlow, Elizabeth Monaghan, Fiona Naylor, and Jonathan Smith (members of the Centre for Mental Health and Safety patient and public involvement and engagement group of the NIHR Greater Manchester Patient Safety Translational Research Centre) for their advisory roles in the study. We would also like to acknowledge all the data providers and general practices that made the anonymised data available for research.

Data sharing

The clinical codes used in this study are available online. The codes are also available from the corresponding author on request. Access to data are available only once approval has been obtained through the individual constituent entities controlling access to the data. The primary care data can be requested via application to the Clinical Practice Research Datalink.

For peer review only - http://bmjopen.bmj.com/site/about/guidelines.xhtml

Table 1. Characteristics of cohorts with a primary care-recorded episode of self-harm in the UK		
	Comparison cohort (2010-2019)	COVID-19 cohort (2020)
Total index episodes (between 10th March and 10th June)	48,739	4,238
Gender:		
Female	29,596 (60.7%)	2,510 (59.2%)
Male	19,143 (39.3%)	1,728 (40.8%)
Age group (yrs):		
10-24	20,308 (41.7%)	1,844 (43.5%)
25-64	25,700 (52.7%)	2,159 (50.9%)
≥65	2,731 (5.6%)	235 (5.6%)
Practice-level IMD quintile:		
1 (least deprived)	5,687 (11.7%)	510 (12.0%)
2	7,003 (14.4%)	600 (14.2%)
3	8,035 (16.5%)	729 (17.2%)
4	10,837 (22.2%)	980 (23.1%)
5 (most deprived)	12,678 (26.0%)	981 (23.2%)
Unknown	4,499 (9.2%)	438 (10.3%)

For peer review only

For peer review only - http://bmjopen.bmj.com/site/about/guidelines.xhtml

Table 2. GP/practice nurse consultations following a primary care-recorded episode of self-harm in the UK

	Face-to-face consultation			Telephone/Video consultation			Face-to-Face or Telephone/Video consultation		
	Comparison cohort, % (n)	COVID-19 cohort, % (n)	Ratio (95% CI)	Comparison cohort, % (n)	COVID-19 cohort, % (n)	Ratio (95% CI)	Comparison cohort, % (n)	COVID-19 cohort, % (n)	Ratio (95% CI)
All persons	80.1 (39,027)	54.3 (2,300)	0.68 (0.66, 0.70)	32.3 (15,723)	67.6 (2,866)	2.10 (2.05, 2.15)	83.2 (40,536)	80.3 (3,403)	0.97 (0.95, 0.98)
Gender:									
Female	82.5 (24,423)	56.3 (1,412)	0.68 (0.66, 0.71)	33.8 (10,004)	69.8 (1,752)	2.06 (2.00, 2.13)	85.5 (25,300)	82.1 (2,060)	0.96 (0.94, 0.98)
Male	76.3 (14,604)	51.4 (888)	0.67 (0.64, 0.71)	29.9 (5,719)	64.5 (1,114)	2.16 (2.07, 2.25)	79.6 (15,236)	77.7 (1,343)	0.98 (0.95, 1.00)
Age group (yrs):									
10-24	74.3 (15,083)	48.2 (888)	0.65 (0.62, 0.68)	24.9 (5,055)	60.5 (1,116)	2.43 (2.33, 2.54)	77.4 (15,720)	74.7 (1,377)	0.96 (0.94, 0.99)
25-64	84.0 (21,596)	58.0 (1,252)	0.69 (0.67, 0.72)	36.4 (9,341)	72.8 (1,571)	2.00 (1.94, 2.06)	86.9 (22,327)	84.0 (1,813)	0.97 (0.95, 0.99)
≥65	86.0 (2,348)	68.1 (160)	0.79 (0.72, 0.87)	48.6 (1,327)	76.2 (179)	1.57 (1.45, 1.70)	91.1 (2,489)	90.6 (213)	0.99 (0.95, 1.04)
Practice-level IMD quintile:									
1 (least deprived)	82.4 (4,687)	56.9 (290)	0.69 (0.64, 0.74)	37.5 (2,135)	68.0 (347)	1.81 (1.69, 1.94)	86.0 (4,891)	81.0 (413)	0.94 (0.90, 0.98)
2	81.6 (5,711)	50.8 (305)	0.62 (0.58, 0.67)	33.4 (2,340)	70.2 (421)	2.10 (1.97, 2.23)	84.5 (5,918)	80.0 (480)	0.95 (0.91, 0.99)
3	81.3 (6,534)	55.8 (407)	0.69 (0.64, 0.73)	34.0 (2,731)	66.3 (483)	1.95 (1.84, 2.07)	84.7 (6,807)	80.0 (583)	0.94 (0.91, 0.98)
4	79.5 (8,613)	53.7 (526)	0.68 (0.64, 0.72)	32.0 (3,472)	67.5 (661)	2.11 (2.00, 2.22)	82.6 (8,949)	80.8 (792)	0.98 (0.95, 1.01)
5 (most deprived)	79.1 (10,023)	56.2 (551)	0.71 (0.67, 0.75)	29.3 (3,717)	68.3 (670)	2.33 (2.21, 2.45)	81.7 (10,358)	81.9 (803)	1.00 (0.97, 1.03)
Unknown	76.9 (3,459)	50.5 (221)	0.66 (0.60, 0.72)	29.5 (1,328)	64.8 (284)	2.20 (2.02, 2.39)	80.3 (3,613)	75.8 (332)	0.94 (0.89, 1.00)

For peer review only

Table 3. Referrals to mental health services following a primary care-recorded episode of self-harm in the UK				
		Comparison cohort: % (n/N)	COVID-19 cohort: % (n/N)	Ratio (95% CI)
All persons		6.1 (2959/48,739)	4.2 (179/4238)	0.70 (0.60, 0.81)
Gender:				
	Female	6.4 (1888/29,596)	3.9 (99/2510)	0.62 (0.51, 0.75)
	Male	5.6 (1071/19,143)	4.6 (80/1728)	0.83 (0.66, 1.03)
Age group (years):				
	10-24	6.7 (1364/20,308)	4.2 (77/1844)	0.62 (0.50, 0.78)
	25-64	5.8 (1490/25,700)	4.0 (87/2159)	0.70 (0.56, 0.86)
	≥65	3.8 (105/2731)	6.4 (15/235)	1.66 (0.98, 2.80)
Practice-level IMD quintile:				
	1 (lowest)	7.4 (420/5687)	6.3 (32/510)	0.85 (0.60, 1.20)
	2	6.8 (475/7003)	4.3 (26/600)	0.64 (0.43, 0.94)
	3	6.4 (512/8035)	4.1 (30/729)	0.65 (0.45, 0.93)
	4	5.8 (629/10,837)	3.5 (34/980)	0.60 (0.43, 0.84)
	5 (highest)	4.9 (623/12,678)	3.8 (37/981)	0.77 (0.55, 1.06)

For peer review only

For peer review only - http://bmjopen.bmj.com/site/about/guidelines.xhtml

Table 4. Prescriptions of any type of psychotropic medication and any antidepressant drug following a primary care-recorded episode of self-harm in the UK

	Any psychotropic medication prescription			Any antidepressant prescription		
	Comparison Cohort, % (n)	COVID-19 cohort, % (n)	Ratio (95% CI)	Comparison cohort, % (n)	COVID-19 cohort, % (n)	Ratio (95% CI)
All persons	54.0 (26,317)	54.9 (2,328)	1.02 (0.99, 1.05)	47.7 (23,243)	49.4	1.04 (1.00, 1.07)
Gender:						
Female	53.6 (15,850)	53.9 (1,354)	1.01 (0.97, 1.05)	48.5 (14,338)	49.4	1.02 (0.98, 1.06)
Male	54.7 (10,467)	56.4 (974)	1.03 (0.99, 1.08)	46.5 (8,905)	49.4	1.06 (1.01, 1.12)
Age group (yrs):						
10-24	30.9 (6265)	35.1 (648)	1.14 (1.07, 1.22)	26.7 (5,430)	31.5	1.18 (1.10, 1.26)
25-64	70.8 (18,195)	70.2 (1,515)	0.99 (0.96, 1.02)	63.3 (16,274)	63.8	1.01 (0.97, 1.04)
≥65	68.0 (1857)	70.2 (165)	1.03 (0.95, 1.13)	56.4 (1,539)	58.7	1.04 (0.93, 1.17)
Practice-level IMD quintile:						
1 (least deprived)	54.1 (3078)	53.7 (274)	0.99 (0.91, 1.08)	48.4 (2,752)	49.2 (251)	1.02 (0.93, 1.12)
2	54.0 (3789)	55.0 (330)	1.02 (0.94, 1.10)	47.9 (3,352)	49.5 (297)	1.03 (0.95, 1.13)
3	54.5 (4378)	56.8 (414)	1.04 (0.98, 1.11)	48.3 (3,883)	50.3 (367)	1.04 (0.97, 1.12)
4	54.2 (5872)	55.0 (539)	1.02 (0.96, 1.08)	47.5 (5,152)	49.8 (488)	1.05 (0.98, 1.12)
5 (most deprived)	53.3 (6758)	55.8 (547)	1.05 (0.99, 1.11)	46.9 (5,945)	50.0 (490)	1.07 (1.00, 1.14)
Unknown	54.3 (2442)	51.1 (224)	0.94 (0.86, 1.04)	48.0 (2,159)	46.1 (202)	0.96 (0.86,1.07)

For peer review only

Table 5. Prescriptions of specific psychotropic medication types following a primary care-recorded episode of self-harm in the UK

	Comparison cohort, % (n)	COVID-19 cohort, % (n)	Ratio (95% CI)
Antidepressants:	47.7 (23,243)	49.4 (2,095)	1.04 (1.00, 1.07)
Antipsychotics:	10.8 (5,285)	12.3 (521)	1.13 (1.04, 1.23)
Anxiolytics/hypnotics:	17.6 (8,577)	15.5 (657)	0.88 (0.82, 0.95)
Mood stabilisers:	5.7 (2,763)	5.6 (238)	0.99 (0.87, 1.13)
Stimulants:	0.8 (369)	1.0 (44)	1.37 (1.00, 1.87)

For peer review only

References

1. NICE. The long term care and treatment of self-harm. Clinical Guideline 133.: National Institute of Health and Care Excellence, 2011.
2. Kapur N, Cooper J, O'Connor RC, et al. Non-suicidal self-injury v. attempted suicide: new diagnosis or false dichotomy? *British Journal of Psychiatry* 2013;202(5):326-28. doi: 10.1192/bjp.bp.112.116111
3. Williams R, Jenkins D, Ashcroft D, et al. Diagnosis of physical and mental health conditions in primary care during the COVID-19 emergency: a retrospective cohort study in a deprived UK city. *Lancet Public Health* 2020 23/09/2020.
4. Carr MJ, Steeg S, Webb RT, et al. Effects of the COVID-19 pandemic on primary care-recorded mental illness and self-harm episodes in the UK: a population-based cohort study. *The Lancet Public health* 2021 doi: 10.1016/s2468-2667(20)30288-7
5. Pierce M, Hope H, Ford T, et al. Mental health before and during the COVID-19 pandemic: a longitudinal probability sample survey of the UK population. *The lancet Psychiatry* 2020 doi: 10.1016/s2215-0366(20)30308-4
6. O'Connor RC, Wetherall K, Cleare S, et al. Mental health and wellbeing during the COVID-19 pandemic: longitudinal analyses of adults in the UK COVID-19 Mental Health & Wellbeing study. *The British journal of psychiatry : the journal of mental science* 2020:1-17. doi: 10.1192/bjp.2020.212
7. Centre for Mental Health. How mental health charities are responding to Covid-19 *https://wwwcentreformentalhealthorguk/sites/default/files/publication/download/CentreforMentalHealth_SectorVoicePaperpdf* 2020.
8. Holland KM, Jones C, Vivolo-Kantor AM, et al. Trends in US Emergency Department Visits for Mental Health, Overdose, and Violence Outcomes Before and During the COVID-19 Pandemic. *JAMA psychiatry* 2021 doi: 10.1001/jamapsychiatry.2020.4402
9. John A, Webb R, Okolie C, et al. The impact of the COVID-19 pandemic on self-harm and suicidal behaviour: update of living systematic review [version 2; peer review: 1 approved, 2 approved with reservations] . *F1000Research* 2021; 9(1097). https://doi.org/10.12688/f1000research.25522.2 (accessed 10.08.2021).
10. John A, DelPozo-Banos M, Gunnell D, et al. Contacts with primary and secondary healthcare prior to suicide: case-control whole-population-based study using person-level linked routine data in Wales, UK, 2000-2017. *The British journal of psychiatry : the journal of mental science* 2020:1-8. doi: 10.1192/bjp.2020.137
11. Carr MJ, Ashcroft DM, Kontopantelis E, et al. Clinical management following self-harm in a UK-wide primary care cohort. *Journal of Affective Disorders* 2016;197:182-88. doi: 10.1016/j.jad.2016.03.013
12. Samaritans. Pushed from pillar to post: Improving the availability and quality of support after self-harm in England. 2020.
13. Mughal F, Troya M, Dikomitis L, et al. Role of the GP in the management of patients with self-harm behaviour: a systematic review. *British Journal of General Practice* 2020;70(694):E364-E73. doi: 10.3399/bjgp20X708257
14. Mroz G, Papoutsi C, Rushforth A, et al. Changing media depictions of remote consulting in COVID-19: analysis of UK newspapers. *British Journal of General Practice* 2021;71(702):E1-E9. doi: 10.3399/bjgp.2020.0967
15. Watts G. COVID-19 and the digital divide in the UK. *Lancet Digital Health* 2020;2(8):E395-E96.
16. NICE. Self-harm: Longer-term management. Evidence update April 2013. Evidence update 39: National Collaborating Centre for Mental Health, 2013.
17. BBC News. Coronavirus: PM says everyone should avoid office, pubs and travelling 2020 [Available from: https://www.bbc.co.uk/news/uk-51917562 accessed 15.02.2021.

For peer review only

18. Wolf A, Dedman D, Campbell J, et al. Data resource profile: Clinical Practice Research Datalink (CPRD) Aurum. *International Journal of Epidemiology* 2019;48(6):1740-+. doi: 10.1093/ije/dyz034
19. Herrett E, Gallagher AM, Bhaskaran K, et al. Data Resource Profile: Clinical Practice Research Datalink (CPRD). *International Journal of Epidemiology* 2015;44(3):827-36. doi: 10.1093/ije/dyv098
20. Padmanabhan S, Carty L, Cameron E, et al. Approach to record linkage of primary care data from Clinical Practice Research Datalink to other health-related patient data: overview and implications. *European Journal of Epidemiology* 2019;34(1):91-99. doi: 10.1007/s10654-018-0442-4
21. Ministry of Housing CaLG. English indices of deprivation 2019. 2020 [Available from: https://www.gov.uk/government/statistics/english-indices-of-deprivation-2019.
22. NHS Digital. SNOMED CT 2021 [Available from: https://digital.nhs.uk/services/terminology-and-classifications/snomed-ct accessed 02.11.2021.
23. NHS Digital. UK Read Code 2015 [Available from: https://data.gov.uk/dataset/f262aa32-9c4e-44f1-99eb-4900deada7a4/uk-read-code.
24. Benchimol EI, Smeeth L, Guttmann A, et al. The REporting of studies Conducted using Observational Routinely-collected health Data (RECORD) Statement. *Plos Medicine* 2015;12(10) doi: 10.1371/journal.pmed.1001885
25. Mold F, Hendy J, Lai Y-L, et al. Electronic Consultation in Primary Care Between Providers and Patients: Systematic Review. *Jmir Medical Informatics* 2019;7(4):4-17. doi: 10.2196/13042
26. Kooth. DATA RELEASE JULY 06 2020. Week 16: How Covid-19 is Affecting The Mental Health of Children and Young People (CYP). 2020 [Available from: https://xenzone.com/wp-content/uploads/2020/07/Week16CYPv2.pdf.
27. Samaritans. Coronavirus, young people and self-harm 2020 [Available from: https://www.samaritans.org/about-samaritans/research-policy/understanding-our-callers-during-covid-19-pandemic/coronavirus-young-people-and-self-harm/.
28. Armitage R. Antidepressants, primary care, and adult mental health services in England during COVID-19. *The lancet Psychiatry* 2021;8(2):e3-e3. doi: 10.1016/s2215-0366(20)30530-7
29. National Collaborating Central for Mental Health. The Community Mental Health Framework for Adults and Older Adults. *https://wwwenglandnhsuk/wp-content/uploads/2019/09/community-mental-health-framework-for-adults-and-older-adultspdf* 2019.

For peer review only

Supplementary material

Table S1: The RECORD statement – checklist of items, extended from the STROBE statement, that should be reported in observational studies using routinely collected health data.

	Item No.	STROBE items	Location in manuscript where items are reported	RECORD items	Location in manuscript where items are reported
**Title and abstract**					
	1	(a) Indicate the study's design with a commonly used term in the title or the abstract (b) Provide in the abstract an informative and balanced summary of what was done and what was found	Title and abstract (page 1 and 2)	RECORD 1.1: The type of data used should be specified in the title or abstract. When possible, the name of the databases used should be included.<br><br>RECORD 1.2: If applicable, the geographic region and timeframe within which the study took place should be reported in the title or abstract.<br><br>RECORD 1.3: If linkage between databases was conducted for the study, this should be clearly stated in the title or abstract.	Abstract: 'Design', 'Setting' and 'Outcome measures' (page 2)<br><br>Abstract: 'Participants', (page 2)<br><br>N/A
**Introduction**					
Background rationale	2	Explain the scientific background and rationale for the investigation being reported			Introduction: paragraphs 1-4 (pages 4-5)
Objectives	3	State specific objectives, including any prespecified hypotheses			Introduction: paragraph 5 (page 5)
**Methods**					

For peer review only

Study Design	4	Present key elements of study design early in the paper			Introduction, paragraph 5 (page 5) and Methods: paragraph 1 '*Study design, data sources, and participants*' (page 6)
Setting	5	Describe the setting, locations, and relevant dates, including periods of recruitment, exposure, follow-up, and data collection			Methods, paragraph 1: '*Study design, data sources, and participants*' (page 6)
Participants	6	*(a) Cohort study* - Give the eligibility criteria, and the sources and methods of selection of participants. Describe methods of follow-up<br>*Case-control study* - Give the eligibility criteria, and the sources and methods of case ascertainment and control selection. Give the rationale for the choice of cases and controls<br>*Cross-sectional study* - Give the eligibility criteria, and the sources and methods of selection of participants	Methods, paragraph 2: '*Study design, data sources, and participants*' (page 6) and paragraph 3: '*Exposures, outcomes and covariates*' (page 7)	RECORD 6.1: The methods of study population selection (such as codes or algorithms used to identify subjects) should be listed in detail. If this is not possible, an explanation should be provided.<br><br>RECORD 6.2: Any validation studies of the codes or algorithms used to select the population should be referenced. If validation was conducted for this study and not published elsewhere, detailed methods and results should be provided.	Methods, paragraph 2: '*Study design, data sources, and participants*' (page 6) and paragraph 3: '*Exposures, outcomes and covariates*' (page 7)<br><br>N/A

For peer review only - http://bmjopen.bmj.com/site/about/guidelines.xhtml

		(b) Cohort study - For matched studies, give matching criteria and number of exposed and unexposed<br>*Case-control study* - For matched studies, give matching criteria and the number of controls per case	N/A	RECORD 6.3: If the study involved linkage of databases, consider use of a flow diagram or other graphical display to demonstrate the data linkage process, including the number of individuals with linked data at each stage.	N/A
Variables	7	Clearly define all outcomes, exposures, predictors, potential confounders, and effect modifiers. Give diagnostic criteria, if applicable.	Methods, paragraph 3: '*Exposures, outcomes and covariates*' (page 7)	RECORD 7.1: A complete list of codes and algorithms used to classify exposures, outcomes, confounders, and effect modifiers should be provided. If these cannot be reported, an explanation should be provided.	Methods, paragraph 3: '*Exposures, outcomes and covariates*' (page 7)
Data sources/ measurement	8	For each variable of interest, give sources of data and details of methods of assessment (measurement).<br>Describe comparability of assessment methods if there is more than one group	Methods, paragraph 2: '*Study design, data sources, and participants*' (page 6) and paragraph 3: '*Exposures, outcomes and covariates*' (page 7)		Methods, paragraph 2: '*Study design, data sources, and participants*' (page 6) and paragraph 3: '*Exposures, outcomes and covariates*' (page 7)
Bias	9	Describe any efforts to address potential sources of bias			Methods, paragraph 2: '*Study design, data sources, and participants*' (page 6)

For peer review only

For peer review only - http://bmjopen.bmj.com/site/about/guidelines.xhtml

Study size	10	Explain how the study size was arrived at			Methods, paragraph 2: '*Study design, data sources, and participants'* (page 6)
Quantitative variables	11	Explain how quantitative variables were handled in the analyses. If applicable, describe which groupings were chosen, and why			Methods, paragraph 2: '*Study design, data sources, and participants'* (page 6)
Statistical methods	12	(a) Describe all statistical methods, including those used to control for confounding<br>(b) Describe any methods used to examine subgroups and interactions			Methods, paragraph 3: '*Exposures, outcomes and covariates'* (page 7) paragraph 4: 'Analysis' (page 7)
		(c) Explain how missing data were addressed			Methods, paragraph 2: '*Study design, data sources, and participants'* (page 6)
		(d) *Cohort study* - If applicable, explain how loss to follow-up was addressed			Methods, paragraph 2: '*Study design, data sources, and participants'*

For peer review only - http://bmjopen.bmj.com/site/about/guidelines.xhtml

		Case-control study - If applicable, explain how matching of cases and controls was addressed<br>*Cross-sectional study* - If applicable, describe analytical methods taking account of sampling strategy<br>(e) Describe any sensitivity analyses			(page 6)<br><br>N/A
Data access and cleaning methods		..		RECORD 12.1: Authors should describe the extent to which the investigators had access to the database population used to create the study population.<br><br>RECORD 12.2: Authors should provide information on the data cleaning methods used in the study.	'Author contributions', (page 11)<br><br>Methods, paragraph 3: '*Exposures, outcomes and covariates'* (page 7)
Linkage		..		RECORD 12.3: State whether the study included person-level, institutional-level, or other data linkage across two or more databases. The methods of linkage and methods of linkage quality evaluation should be provided.	Methods: '*Study design, data sources, and participants'* (page 6)
**Results**					
Participants	13	(a) Report the numbers of individuals at each stage of the study (*e.g.*, numbers potentially eligible, examined for eligibility, confirmed eligible, included in the		RECORD 13.1: Describe in detail the selection of the persons included in the study (*i.e.,* study population selection) including filtering based on data quality, data availability and linkage. The selection of included persons can be	Results: paragraph 1 (page 8)

For peer review only

		study, completing follow-up, and analysed)<br>(b) Give reasons for non-participation at each stage.<br>(c) Consider use of a flow diagram		described in the text and/or by means of the study flow diagram.	
Descriptive data	14	(a) Give characteristics of study participants (*e.g.*, demographic, clinical, social) and information on exposures and potential confounders<br>(b) Indicate the number of participants with missing data for each variable of interest<br><br>(c) *Cohort study* - summarise follow-up time (*e.g.*, average and total amount)			Results: paragraph 1 (page 8)<br><br>Methods, paragraph 2: '*Study design, data sources, and participants*' (page 6)<br><br>Methods, Paragraph 2: '*Study design, data sources, and participants*' (page 6)
Outcome data	15	*Cohort study* - Report numbers of outcome events or summary measures over time<br>*Case-control study* - Report numbers in each exposure category, or summary measures of exposure			Table 1 (page 13)

For peer review only

		Cross-sectional study - Report numbers of outcome events or summary measures			
Main results	16	(a) Give unadjusted estimates and, if applicable, confounder-adjusted estimates and their precision (e.g., 95% confidence interval). Make clear which confounders were adjusted for and why they were included<br>(b) Report category boundaries when continuous variables were categorized<br>(c) If relevant, consider translating estimates of relative risk into absolute risk for a meaningful time period			Results, paragraphs 1-4: (page 8) and Tables 1-4 (Pages 13-16)
Other analyses	17	Report other analyses done—e.g., analyses of subgroups and interactions, and sensitivity analyses			Results, paragraph 1: (page 8) and Tables 1-4 (Pages 13-16)
**Discussion**					
Key results	18	Summarise key results with reference to study objectives			Discussion, paragraph 1: 'Summary' (Page 9)
Limitations	19	Discuss limitations of the study, taking into account sources of potential bias or imprecision. Discuss both direction and magnitude of any potential bias	Discussion, paragraph 2: 'Strengths and limitations' (page 9-10)	RECORD 19.1: Discuss the implications of using data that were not created or collected to answer the specific research question(s). Include discussion of misclassification bias, unmeasured confounding, missing data, and changing	Discussion, paragraph 2: 'Strengths and limitations' (page 9-10)

For peer review only

				eligibility over time, as they pertain to the study being reported.	
Interpretation	20	Give a cautious overall interpretation of results considering objectives, limitations, multiplicity of analyses, results from similar studies, and other relevant evidence			Discussion, paragraphs 1-2: 'Implications for research and practice' (page 10-11)
Generalisability	21	Discuss the generalisability (external validity) of the study results			Discussion, paragraph 2: 'Strengths and limitations' (page 9-10)
**Other Information**					
Funding	22	Give the source of funding and the role of the funders for the present study and, if applicable, for the original study on which the present article is based			Funding statement (page 11)
Accessibility of protocol, raw data, and programming code		..		RECORD 22.1: Authors should provide information on how to access any supplemental information such as the study protocol, raw data, or programming code.	Data sharing statement (Page 12)

*Reference: Benchimol EI, Smeeth L, Guttmann A, Harron K, Moher D, Petersen I, Sørensen HT, von Elm E, Langan SM, the RECORD Working Committee. The REporting of studies Conducted using Observational Routinely-collected health Data (RECORD) Statement. *PLoS Medicine* 2015; in press.

*Checklist is protected under Creative Commons Attribution (CC BY) license.

For peer review only
